# Post-COVID-19 pandemic organ donation activities in Germany: a multicenter retrospective analysis

**DOI:** 10.3389/fpubh.2024.1356285

**Published:** 2024-02-20

**Authors:** Jan Sönke Englbrecht, Daniel Schrader, Jörg Benedikt Alders, Melanie Schäfer, Martin Soehle

**Affiliations:** ^1^Department of Anesthesiology, Intensive Care and Pain Medicine, University Hospital Münster, Münster, Germany; ^2^The Medical Director's Staff Division of Organ Donation Coordination, University Hospital Düsseldorf, Düsseldorf, Germany; ^3^Department of Anesthesiology, Intensive Care Medicine and Pain Therapy, University Hospital Knappschaftskrankenhaus, Ruhr University Bochum, Bochum, Germany; ^4^Department of Intensive Care Medicine, University Hospital Rheinisch-Westfälische Technische Hochschule (RWTH) Aachen, Aachen, Germany; ^5^Department of Anesthesiology and Intensive Care Medicine, University Hospital Bonn, Bonn, Germany

**Keywords:** COVID-19, organ donation, brain death, conversion rate, organ procurement, ICU capacity

## Abstract

**Introduction:**

The COVID-19 pandemic had a negative impact on the number of solid organ transplantations. After a global decline of 16% in 2020, their numbers subsequently returned to pre-pandemic levels. In contrast, numbers in Germany remained almost constant in 2020 and 2021 but fell by 6.9% in 2022. The reasons for this divergent development are unknown.

**Methods:**

The number of deceased with a severe brain damage, potential and utilized donors after braindeath and the intensive care unit treatment capacity were retrospectively compared for the years 2022 and 2021 at five university hospitals in North Rhine-Westphalia, Germany. Reasons for a donation not utilized were reviewed. To enable a comparison of the results with the whole of Germany and the pre-pandemic period, numbers of potential and utilized donors were extracted from official organ donation activity reports of all harvesting hospitals in Germany for the years 2019–2022.

**Results:**

The numbers of deceased with a severe brain damage (−10%), potential (−9%), and utilized donors after braindeath (−44%), and intensive care unit treatment capacities (−7.2%) were significantly lower in 2022 than 2021. A COVID-19 infection was a rarer (−79%), but donor instability (+44%) a more frequent reason against donation in 2022, whereas preserved brain stem reflexes remained the most frequent reason in both years (54%). Overall numbers of potential and utilized donations in Germany were lower in 2022 than in the pre-pandemic period, but this was mainly due to lower numbers in hospitals of lower care. The number of potential donors in all university hospitals were higher in 2022 but utilized donations still lower than in 2019.

**Conclusion:**

The decrease in potential and utilized donations was a result of reduced intensive care unit treatment capacities and a lower conversion rate at the five university hospitals. A COVID-19 infection did not play a role in 2022. These results indicate that ICU treatment capacities must be restored to increase donations. The lower number of potential donors and the even lower conversion rate in 2022 throughout Germany show that restructuring the organ procurement process in Germany needs to be discussed to increase the number of donations.

## 1 Introduction

The COVID-19 pandemic led to restrictions in healthcare worldwide, especially at the beginning of the pandemic situation. In Germany, the first restrictions were announced in March 2020 as part of a pandemic-related lockdown, affecting healthcare services (1). The hospital admission rates were substantially reduced following this national lockdown (2). Even a notable reduction in emergency procedures and time critical interventions was detectable (3–6). This was, among other things, the result of reduced outpatient examinations, closed medical practices, avoidance of hospitalization out of concern for infection with COVID-19 and reduced capacities of intensive care units (ICU) (6–8).

The pandemic also had a major global impact on solid organ transplantation. An estimated 16% global reduction in transplant activity occurred over the course of 2020 (9). In the Eurotransplant (ET) region (Germany is a member of ET), an 11.6% decline in donations after braindeath (DBD) was found between March 2020 and February 2021 (10). Concerns about potential donor-to-recipient transmission, post-transplant management, ethics and legal issues caused great uncertainty (10). Restrictions on the criteria for eligible donors may have led to a further reduction in organ donations (11). With ICU bed and staff shortages, donor evaluation could not always be accommodated (9). Limited possibilities to visit relatives in the hospital could have reduced opportunities for broaching organ donation with families (12), and consent rate was negatively affected in some countries (13). From 2021 on, DBD were returning to prepandemic levels (9, 14). In the ET region, DBD increased by 1.3 and 6.9% in 2021 and 2022, respectively (10, 15). In contrast, DBD in Germany decreased by 6.9% in 2022, with a significant drop in the first quarter (16).

The German organ procurement organization (Deutsche Stiftung Organtransplantation—DSO) explains this decline with the COVID-19 pandemic and the subsequent shortage of healthcare services (16). However, DBD did not substantially decrease in Germany in 2020 (−2.0%) and increased in 2021 (+2.2%), despite the pandemic situation already prevailing at that time with significantly higher admissions of COVID-19 patients to the ICU than 2022 (17, 18). Consequently, it seems questionable, that the decline in 2022 in Germany was still a result of the pandemic situation. On the other hand, the cumulative number of unoccupied ICU-beds in Germany was still 12% lower in 2022 compared to 2021 (19), indicating that ICU treatment capacities continue to be reduced despite declining COVID-19 admissions.

Potential DBD donors [defined as a patient with a devastating brain injury or lesion whose clinical condition is suspected to fulfill braindeath criteria (20)] are recruited from the cohort of mechanically ventilated ICU-patients with a severe brain damage. The course of the disease does not allow postponement of admission, diagnosis, and therapy, especially since these patients usually cannot decide for themselves whether they will be admitted to a hospital (21). Reduced ICU capacities during the pandemic may therefore play a rather minor role in the admission of potential DBD donors (pDBD). As information on the total number of pDBD in Germany is generally sparse (22), and available studies focused on the number of utilized DBD donors (uDBD) rather than pDBD (23), the impact of the pandemic-related healthcare restrictions on the number of pDBD remains unknown.

Studies from the first wave of the pandemic in Germany could show reduced admissions of patients with a severe brain damage, e.g., due to stroke (24, 25), or neurosurgical emergencies (26–28), presumably reducing the number of pDBD at that time. Especially smaller hospitals had substantially reduced ICU capacities, resulting in more referrals to higher care level hospitals (21). Patient referrals, delayed admission, and treatment due to reduced ICU capacity potentially worsens outcome in patients with severe brain damage. Hospitals of higher care in Germany reported increased mortality rates for aneurysmal subarachnoid hemorrhage and a dramatic increase in neurovascular cases during the first wave of the pandemic (29, 30), as well as for in-hospital mortality after acute ischemic stroke (24). The pre-hospital rescue time for trauma patients was prolonged (31) and neurotrauma emergencies increased (21). Therefore, worsened outcome after severe brain damage due to limited treatment capacity could increase the number of pDBD, at least in tertiary care or university hospitals. Since nearly 80% of all uDBD in Germany originate from these type of hospitals (32), the question arises, whether the 2022 decline in organ donation in Germany was due to reduced ICU capacities, a changed number of pDBD or a reduced conversion of a potentially higher number of pDBD into uDBD, and whether this development differed depending on the type of hospital.

In this study, we compared the number of pDBD, uDBD, reasons for a pDBD not utilized, and ICU treatment capacities in 2022 with 2021 at five university hospitals in the federal state of North Rhine-Westphalia (NRW), Germany. The aim was to find out more about the reasons for the decline in uDBD in Germany in 2022 and a possible influence of the COVID-19 pandemic. In addition, the results from this period were related to official numbers about post-mortem organ donation activities in all harvesting hospitals in Germany for the period before (2019), during the first phase (2020) and the following years (2021 and 2022) of the pandemic.

## 2 Methods

This retrospective observational study was approved by the Ethics Committee of the University of Muenster on July 18, 2023 (File Number 2021-801-f-S).

**Figure 1:**
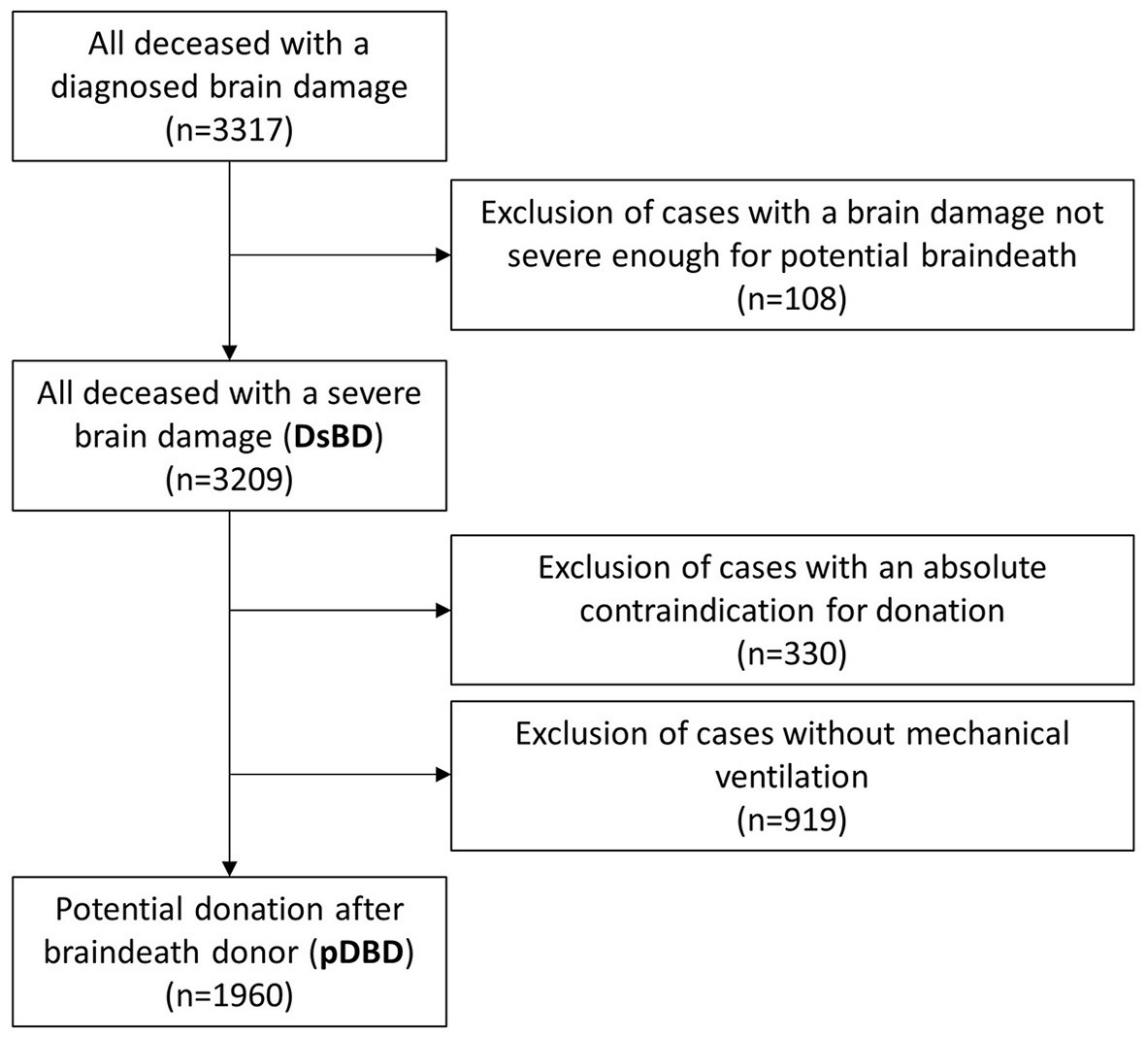
Flow chart of the selection process.

In a first step, all patients, who were treated at the University Hospital Münster (UKM), University Hospital Düsseldorf (UKD), University Hospital Bochum (UKRUB), University Hospital Aachen (UKA), and University Hospital Bonn (UKB) between January 2021 and December 2022, were retrospectively screened for in-hospital death and a diagnosed brain damage. This information was extracted from the patient data according to § 21 Hospital Remuneration Act [a law that legally regulates the charges for full and partial inpatient hospital services in Germany (33)]. All identified cases were subjected to a selection procedure, which was adapted from a method previously described (34, 35), to identify deceased with brain damage that was considered severe enough to potentially progress to braindeath (deceased with severe brain damage—DsBD). The resulting number of pDBD was obtained after excluding the cases in which there was an absolute contraindication against organ donation, or which were never mechanically ventilated (Figure 1). The subsequent individual case analysis of all pDBD identified by this selection procedure was performed by reviewing the medical record files. Cases were categorized based on utilized donations or the documented reason against a donation (preserved brain stem reflexes, medical contraindications (e.g., malignancy and infection), COVID-19 infection, severe organ dysfunction, refused consent to donate). If two or more reasons applied for categorization against donation (e.g., preserved brainstem reflexes and refused consent), the case was assigned to the category originally documented in the medical record file as the main reason against donation. The numbers for 2021 and 2022 were compared and the conversion rate (uDBD divided by pDBD) was calculated for both years.

In the second step, the average monthly ICU occupancy days (ICU-OD) of the participating five university hospitals in NRW [UKM, UKD, UKRUB, UKA, and UKB - called UH-NRW in the following] were retrospectively evaluated for the years 2021 and 2022 and additionally for the years 2019 and 2020, to compare numbers for the period before and during the pandemic. The number of ICU-OD was defined as the sum of the fully inpatient ICU-patients of each day at 12 p.m. (sum of midnight stock) (36). The ICU-OD were used as a marker for the ICU treatment capacity, because these numbers are more reliable than a theoretical capacity of existing ICU beds that might not be fully operable because of staff shortage. The in-house medical controlling departments provided mean numbers for every month from January 2019 to December 2022.

In the third step, monthly numbers of ICU-OD in 2021 and 2022 and the corresponding numbers of DsBD were correlated.

Finally, the annual reports about donation activities of all harvesting hospitals in Germany (organ donation reports—OD-reports) between 2019 and 2022 were reviewed. Harvesting hospitals in Germany have a legal obligation to provide data for OD-reports, which are prepared and published by the DSO (32). The OD-reports include information about the total number of deceased with a brain damage, DsBD, pDBD and uDBD, respectively. The DSO prepares these numbers for all harvesting hospitals in Germany in total and additionally separated by regions in Germany (seven regions in total, with NRW being the largest region in terms of population) and the provided level of care (level A: university hospitals, level B: hospitals with neurosurgery department, level C: hospitals without neurosurgery department). The algorithm used by the DSO to generate these numbers was extensively validated in several studies (33, 37, 38) and showed to be very sensitive in detecting DsBD but to lack specificity in identifying pDBD (34). The annual numbers of DsBD, pDBD and uDBD from 2019 to 2022 were extracted from the OD-reports for three groups:

- all harvesting hospitals in NRW
- all harvesting hospitals in Germany
- all harvesting hospitals separated by their level of care (level A, level B, level C)

This was an exploratory study and not based on a formal power calculation. Statistical analysis was performed using SPSS (IBM company, version 28). A one-way multivariate analysis of variance (MANOVA) was used to compare the years 2021 and 2022 and dependent variables (deceased with brain damage, brain damage classified not severe enough for potential braindeath, DsBD, absolute contraindications, not mechanically ventilated, pDBD, age of pDBD, uDBD, reasons against donation, and ICU-OD). *Post-hoc* univariate ANOVAs were conducted for every dependent variable. ICU-OD from 2019 to 2022 and quarterly numbers for 2021 and 2022 were analyzed using one-factor ANOVA, with Turkey *post-hoc* analyses. The calculation of correlation coefficients was done according to Pearson and interpreted according to Cohen. A *p*-value ≤ 0.05 was defined significant.

## 3 Results

### 3.1 Identification of DsBD and pDBD

A total of 3,317 deceased with a diagnosed brain damage were identified between January 2021 and December 2022 at the UH-NRW. One hundred and eight cases were excluded, because brain damage was not classified as severe enough to potentially lead to braindeath, resulting in 3,209 DsBD. After excluding 330 cases because of an absolute contraindication against donation and 919 cases without mechanical ventilation, 1,960 pDBD remained for further analysis and 75 donations were utilized (Figure 1; Table 1).

**Table 1:** Demographics of the study cohort.

	**Deceased with brain damage (** ***n*** **)**		**Brain damage not classified as severe (** ***n*** **)**		**DsBD (** ***n*** **)**		**Potential DBD [Age (mean** ±**SD)]**		**Utilized DBD (** ***n*** **)**	
**Year**	**2021**	**2022**	**2021**	**2022**	**2021**	**2022**	**2021**	**2022**	**2021**	**2022**
UKA	383	302	12	8	371	294	213 (72.1 ± 2.4)	186 (69.5 ± 4.0)	5	2
UKB	477	420	6	9	471	411	279 (69.0 ± 4.2)	232 (68.2 ± 3.3)	12	5
UKD	375	345	30	25	345	320	237 (66.7 ± 3.1)	212 (66.0 ± 4.0)	13	10
UKM	317	304	7	5	310	299	187 (68.9 ± 5.8)	193 (65.5 ± 5.3)	10	5
UK RUB	195	199	2	4	193	195	112 (71.7 ± 4.5)	109 (74.0 ± 4.0)	8	5
Total	1,747	1,570	57	51	1,690	1,519	1,028 (69.7 ± 2.4)	932 (68.6 ± 2.7)	48	27

DsBD, Deceased with severe brain damage; SD, standard deviation; DBD, donation after braindeath; UKA, University Hospital Aachen; UKB, University Hospital Bonn; UKD, University Hospital Düsseldorf; UKM, University Hospital Münster; UKRUB, University Hospital Bochum.

A one-way MANOVA showed significant differences between 2021 and 2022 on the dependent variables, *F*_(4,19)_ = 5.162, *p* = 0.005, partial η^2^ = 0.521, Wilk's Λ = 0.479 (Table 2). *Post-hoc* analysis revealed a significant decrease in the number of deceased with a brain damage (−10%), DsBD (−10%), absolute contraindications for a donation (−34%) and the resulting numbers of pDBD (−9%) in 2022.

**Table 2:** Number of identified patients in the different selection levels.

**Selection level**	**2021**	**2022**	**2022/2021 (%)**	***p***
Deceased with brain damage	1,747	1,570	−10	0.005
Brain damage considered not severe enough to result in braindeath	57	51	−11	0.644
Deceased with severe brain damage (DsBD)	1,690 (100%)	1,519 (100%)	−10	0.004
Absolute CI for DBD	199 (12%)	131 (9%)	−34	< 0.001
Not mechanically ventilated	463 (27%)	456 (30%)	−2	0.809
Potential DBD donor (pDBD)	1,028 (61%)	932 (61%)	−9	0.028

One-way MANOVA was used for differences between years and the dependent variables. Post-hoc univariate ANOVAs were conducted for every dependent variable. Numbers in brackets show proportion.

CI, contraindication; DBD, donation after braindeath.

### 3.2 Utilized donations and reasons against donation in pDBD

A one-way MANOVA showed significant difference between 2021 and 2022 on the dependent variables, *F*_(8,15)_ = 5.641, *p* = 0.002, partial η^2^ = 0.751, Wilk's Λ = 0.249 (Table 3). Mean age of pDBD at UH-NRW was 69.7 in 2021 and 68.6 in 2022, respectively. Preserved brain stem reflexes was the most frequent reason against uDBD and proportion remained unchanged between both years (54%), as well as medical contraindications. A COVID-19 infection was significantly rarer (−79%), and severe organ dysfunction a significantly more frequent reason (+44%) against a donation in 2022. Refused consent was lower in 2022 (−16%) without reaching statistical significance. The resulting number of uDBD decreased significantly by 44% in 2022. Accordingly, ICU-therapy was withdrawn without a utilized donation in 95% of pDBD in 2021 and 97% in 2022, respectively. ICU-OD decreased significantly in 2022 (−7%) compared to 2021 (Table 3). Conversion rate (uDBD divided by pDBD) decreased from 4.7% in 2021 to 2.9% in 2022.

**Table 3:** Reasons against donation, utilized donations, and ICU capacity.

**pDBD**	**2021 (*n* = 1,028)**	**2022 (*n* = 932)**	**2022/2021 (%)**	***p***
Age (mean ± SD)	69.7 ± 2.4	68.6 ± 2.7		0.335
Preserved brain stem reflexes	553 (54%)	503 (54%)	−9	0.060
Medical CI (e.g., tumor and infection)	115 (11%)	123 (13%)	+7	0.665
COVID-19 infection	67 (7%)	14 (2%)	−79	0.015
Severe organ dysfunction	97 (9%)	140 (15%)	+44	0.014
Refused consent	148 (14%)	125 (13%)	−16	0.190
uDBD	48 (5%)	27 (3%)	−44	0.014
Monthly ICU-OD (mean ± SD)	13,156 ± 470	12,215 ± 470	−7	< 0.001

One-way MANOVA was used for differences between years and the dependent variables. Post-hoc univariate ANOVAs were conducted for every dependent variable. Numbers in brackets show proportion.

pDBD, potential donation after braindeath donor; SD, standard deviation; CI, contraindication; uDBD, utilized donation after braindeath donor; ICU-OD, intensive care unit occupancy days.

### 3.3 Intensive care unit treatment capacity from 2019 to 2022

ICU treatment capacity (as measured by ICU-OD) of the UH-NRW differed statistically significant between 2019 and 2022 (*p* < 0.001). *Post-hoc* analysis revealed a significant decrease of the ICU-OD for 2022 compared to 2021 (−7.2%), and for 2021 and 2022 compared to 2019, respectively (Table 4).

**Table 4:** Intensive care unit occupancy days at the University Hospitals of Aachen, Bochum, Bonn, Düsseldorf, and Münster.

	**2019**	**2020**	**2021**	**2022**
Monthly ICU occupancy days (mean ± SD)	13,723 ± 436	13,920 ± 438	13,156 ± 470	12,215 ± 470
Difference to previous year		+1.4% (*p* = 0.715)	−5.5% (*p* < 0.001)	−7.2% (*p* < 0.001)
Difference to 2019		+1.4% (*p* = 0.715)	−4.1% (*p* = 0.019)	−11.0% (*p* < 0.001)

A one-way ANOVA was performed to assess the differences between years for ICU occupancy days, with Turkey post-hoc analyses for each year.

ICU, intensive care unit; SD, standard deviation.

### 3.4 Time course of ICU-OD, DsBD, and pDBD

**Figure 2:**
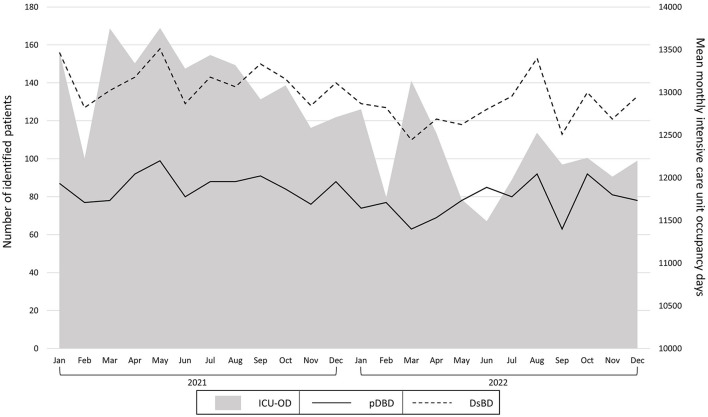
Monthly numbers of deceased with a severe brain damage (DsBD; dotted black line), potential donation after braindeath donors (pDBD; black line) and mean monthly intensive care unit occupancy days (ICU-OD; gray area).

Monthly numbers for DsBD, pDBD, and ICU-OD are shown in Figure 2. One-way ANOVA showed no significant difference for the quarterly numbers of DsBD (*p* = 0.190) and pDBD (*p* = 0.180) between 2021 and 2022. Quarterly numbers of the ICU-OD differed significantly (*p* = 0.007), with a significant decrease in the second quarter of 2022 compared to the second quarter of 2021 [ICU-OD (mean ± SD) Q2 2022/Q2 2021: 11.919 ± 535/13.456 ± 259, *p* = 0.017].

### 3.5 Correlation between ICU-OD and DsBD

**Figure 3:**
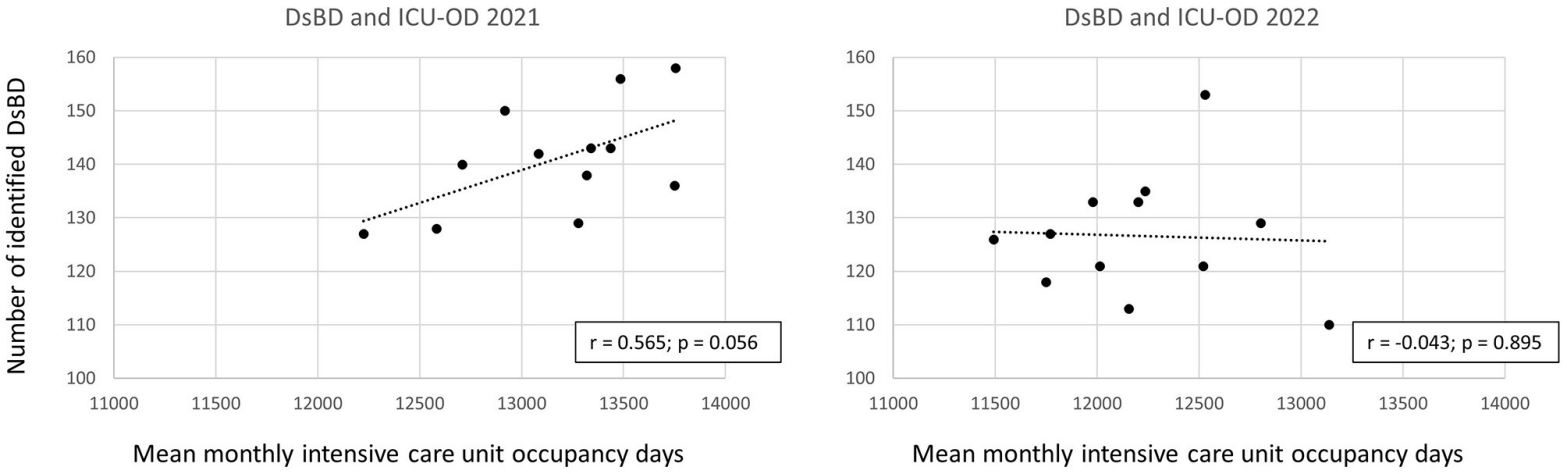
Correlation between the number of deceased with a severe brain damage (DsBD) and intensive care unit occupancy days (ICU-OD). The numbers for 2021 are shown on the left and for 2022 on the right.

Pearson-correlation between ICU-OD and DsBD was strong in 2021, but weak negative in 2022 (Figure 3).

### 3.6 Organ donation activity in Germany from 2019 to 2022

The number of DsBD, pDBD and uDBD from 2019 to 2022, extracted from OD-reports are shown in Table 5. There was a remarkable drop of DsBD, pDBD and uDBD in 2022 compared to 2019 in Germany, which was even more pronounced in NRW. Level A and level B hospitals in Germany showed a comparable drop of DsBD and pDBD in 2020 compared to 2019, while these figures fell less sharply at level C hospitals. From 2020 to 2022, DsBD and pDBD increased again continuously in level A hospitals. Level B hospitals showed fluctuating numbers for this period, whereas a continuous reduction of DsBD and pDBD from 2020 to 2022 was detectable in level C hospitals. The numbers of uDBD showed a variable development for level A, B, and C hospitals. In level A hospitals, uDBD decreased in 2020, increased in 2021 and decreased again in 2022. In level B and level C hospitals, uDBD decreased continuously from 2020 to 2022. Consequently, the share of uDBD originating from level A hospitals increased from 34% in 2019 to 37% in 2022 and decreased continuously in level C hospitals from 25% in 2019 to 22% in 2022.

**Table 5:** Deceased with a severe brain damage, potential, and utilized donors in Germany.

**Hospital**	**2019**	**2020**	**2021**	**2022**
**NRW**				
DsBD	13,817	13,162 (−5%)	12,012 (−13%)	11,276 (−18%)
pDBD	5,377	5,119 (−5%)	4,857 (−10%)	4,371 (−19%)
uDBD	177	168 (−5%)	186 (+5%)	147 (−17%)
**Germany**				
DsBD	61,216	57,603 (−6%)	56,700 (−7%)	54,684 (−11%)
pDBD	23,948	22,363 (−7%)	22,803 (−5%)	21,667 (−10%)
uDBD	910	863 (−5%)	875 (−4%)	794 (−13%)
**Level A hospitals**				
DsBD	10,542	9,699 (−8%)	10,389 (−1%)	10,748 (+2%)
pDBD	5,976	5,342 (−11%)	6,045 (+1%)	6,062 (+1%)
uDBD	306	285 (−7%)	324 (+6%)	291 (−5%)
Proportion of all uDBD in Germany	34%	33%	37%	37%
**Level B hospitals**				
DsBD	18,383	16,969 (−8%)	17,264 (−6%)	17,095 (−7%)
pDBD	8,762	7,828 (−11%)	7,696 (−12%)	7,423 (−15%)
uDBD	377	367 (−3%)	352 (−7%)	326 (−14%)
Proportion of all uDBD in Germany	41%	43%	40%	41%
**Level C hospitals**				
DsBD	32,291	30,935 (−4%)	29,047 (−10%)	26,841 (−17%)
pDBD	9,210	9,193 (0%)	9,062 (−2%)	8,182 (−11%)
uDBD	227	211 (−7%)	199 (−12%)	177 (−22%)
Proportion of all uDBD in Germany	25%	24%	23%	22%

Numbers are taken from the organ donation activity reports of all harvesting hospitals in Germany (32). Numbers in brackets show percentage changes compared to 2019.

NRW, North Rhine-Westphalia; DsBD, deceased with a severe brain damage; pDBD, potential donation after braindeath donor; uDBD, utilized donation after braindeath donor.

## 4 Discussion

The number of DsBD and pDBD and the ICU treatment capacity at the five university hospitals in NRW was significantly lower in 2022 compared to 2021. A positive correlation between ICU treatment capacity and the number of DsBD in 2021 turned into a negative correlation in 2022. The decrease of DsBD, pDBD and the conversion rate resulted in a significantly lower number of utilized donations in 2022. Main reason for a donation not utilized were preserved brain stem reflexes in both years. A COVID-19 infection was a more frequent reason against donation in 2021, whereas donor instability was more frequent in 2022. Overall numbers of DsBD, pDBD and uDBD in Germany were lower in 2022 compared to the pre-pandemic period, mainly due to a marked decrease in hospitals of lower care. In contrast, DsBD and pDBD at university hospitals throughout Germany increased steadily from 2020 on, but not at university hospitals in this study. This suggests that the reduced ICU treatment capacity at the UH-NRW was a key factor for the decline of potential and utilized donations in 2022 in this cohort.

### 4.1 Numbers of deceased with a severe brain damage

The number of DsBD decreased significantly in 2022. Obviously, a higher ICU capacity enables hospitals to treat more patients with a severe brain damage. Accordingly, there was a positive correlation between ICU-OD and DsBD in 2021. Interestingly, this correlation was weak negative in 2022, suggesting that the numbers of monthly treated DsBD was independent of ICU treatment capacities, albeit at an overall lower level in terms of numbers. A possible explanation is the decreasing number of COVID-19 admissions to the ICU, which may have resulted in a shift toward admission of patients with a severe brain damage in 2022 (17, 39), although their absolute numbers were still lower than 2021. University hospitals were thus capable to treat those patients despite decreasing ICU treatment capacities. Or, to put it in other words, for many patients with a severe brain damage, ICU beds could probably only be found in hospitals of a higher level of care in view of the overall reduced ICU capacity in Germany (39).

This assumption is supported by the numbers from the OD-reports, which show, that numbers of DsBD even increased from 2020 to 2022 at level A hospitals in Germany whilst numbers decreased in level C hospitals. It would be interesting to know, if ICU treatment capacity in hospitals of lower care showed a correlation with numbers of DsBD. The numbers from the OD-reports indicate this, as DsBD decreased from 2019 to 2022 in level C hospitals as did overall ICU treatment capacity (17, 19). Reduced ICU treatment capacity and number of DsBD being independent from each other is thus probably only detectable in higher level care hospitals, who must care for more severely ill patients in the face of an overall reduced ICU capacity in Germany. This means on the other hand, that if correlation between ICU treatment capacity and DsBD would be restored at level A hospitals on an overall lower level of capacity, this could result in a further decrease of DsBD and consequently probably also in utilized donations in Germany.

Numbers of DsBD with an absolute contraindication for a donation were significantly lower in 2022. Absolute contraindications include primarily patients with malignancies (40). It is possible that these patients were transferred less frequently to university hospitals because the reduced ICU capacity meant that patients with a poor prognosis were less likely to be accepted for transfer, although this assumption cannot be confirmed by the available data from this study.

### 4.2 Potential and utilized donations after braindeath and reasons against donation

The proportion of pDBD among DsBD remained the same in both years (61%). With significantly reduced overall numbers of DsBD, this resulted in a significant decrease of pDBD in 2022.

Reasons for a donation not utilized differed between the years. A COVID-19 infection as a reason against donation was less frequent in 2022, likely because at the end of April 2022, the German Medical Association no longer stated a COVID-19 infection as an absolute contraindication for an organ donation (41). Additionally, vaccination and protection strategies supported to minimize the effect of COVID-19 on transplantation activities (10), and resilience throughout the entire ICU, organ donation and transplantation services was improved (42).

In contrast, severe organ dysfunction was more frequent in 2022, suggesting that pDBD were in a more serious condition. Probably, ongoing patient referrals in times of reduced ICU capacity and delayed treatment possibilities resulted in patients with an advanced stage of disease being admitted to university hospitals. This aspect was already shown in the first wave of the pandemic in Germany (21, 29, 30), and might explain the increased incidence of donor instability as a reason against utilized donations.

Conversion rate (uDBD divided by pDBD) was very low in 2021 and even lower in 2022 (4.7 and 2.9%, respectively). This low ratio was already shown for Germany in the prepandemic period (22, 33), whereas data from other countries traditionally show higher conversion rates, e.g., 59% in Canada (43), 33% in Australia (44), and 26–35% in the Netherlands (45), respectively. This observation probably partly explains the consistently low performance in utilized donations in Germany, independent of a pandemic situation. Main reason for the low conversion rate in this cohort were preserved brain stem reflexes in 54% of pDBD in both years. Braindeath cannot be diagnosed if brain stem reflexes are preserved, so that DBD was not possible in these cases. This group of pDBD could potentially become donors after cardiac death (DCD), when therapy is discontinued due to an unfavorable prognosis, but DCD is not possible in Germany due to legal regulations (22). It can be assumed, that a significant share of potential donations was thus lost because of these circumstances (22).

Proportion of cases with preserved brain stem reflexes and medical contraindications were both comparable in 2021 and 2022, presumably because these aspects are unlikely to be influenced by a pandemic situation. Overall, these cases accounted for more than two-thirds of cases with missed conversion to donor status.

The third numerically relevant reason against uDBD was refused consent in both years. Consent to donation was reduced during the first wave of the pandemic in some countries (13), probably due to uncertainty and reduced possibilities to discuss a donation with family members (9, 12). Reliable data for consent rates in Germany during the pandemic are lacking (40), but traditionally, refused consent is a relevant reason against donation in Germany (22, 46). In this cohort, refused consent was higher in 2021 than 2022. Presumably, fewer restrictions on kin visits, greater resilience to COVID-19, and reduced pandemic-induced uncertainty led to greater ease in obtaining consent in 2022 (42).

Overall, this analysis showed no clear evidence, that besides reduction in ICU treatment capacity other specific problems related to the COVID-19 pandemic were the cause of the lower conversion rate and the 44% reduction of uDBD in 2022 at the UH-NRW, which was even more pronounced than the 6.9% decline of donations throughout Germany. A COVID-19 infection in pDBD did not play a role in 2022, and consent rate was not inferior compared to 2021. Reduced donations were a consequence of reduced numbers of DsBD and pDBD and an even lower conversion rate than 2021. Theoretically, if the conversion rate would have been comparable to 2021, this would have resulted in 44 uDBD in 2022, equivalent to only an 8% reduction in donations.

Therefore, the low conversion rate is a problem that needs to be discussed. For example, a significant number of potential donors were not utilized in this cohort, because preserved brain stem reflexes prevented a DBD. An implementation of a DCD-program in Germany would possibly be one option to address this problem. At least in other countries, the implementation of a DCD-program led to an increase in donations (47, 48).

Additionally, Germany is one of the few European countries, where consent to donation is still based on an opt-in system (22). In view of the relevant number of cases with refused consent, changing these regulations to an opt-out system should be considered. Studies could show, that countries with an opt-out system can achieve higher donation rates (49, 50). Accordingly, some politicians attempted to address this issue with a legislative proposal to introduce an opt-out system in Germany, but the majority of members of the German parliament voted against it in 2020 (51). As the number of organ donations has not increased since then, politicians have recently made another attempt to introduce an opt-out system with a proposal to amend the German Transplantation Act. However, it has not yet been decided whether this proposal will be implemented this time.

### 4.3 Intensive care unit treatment capacity

Germany has a comparatively high supply of ICU-beds (52, 53), but even before the pandemic, the high number of beds was not accompanied by adequate staff (52). The advantage of having a large number of beds shrinks significantly when looking at the number of beds that can be operated with the available staff. Germany has one of the lowest ratio of nurses per hospital bed (0.78) in Europe (54). For example, Denmark has 2.6 times as many trained nurses and 2.2 times as many physicians per 1,000 inpatient cases compared with Germany (52). Following the onset of the COVID-19 pandemic, an intensive care register (a digital platform for real-time recording of treatment and ICU-bed capacity of about 1,300 hospitals in Germany) was established in March 2020 in Germany (19). This register showed a continuous decline of available ICU beds in Germany from 2020 to 2022. The university hospitals in NRW were additionally burdened by an 11-week strike of nursing staff from May 2 until July 20, 2022 (55). Consequently, ICU-OD at the UH-NRW was lowest in the second quarter of 2022, and recovered thereafter, but without reaching numbers of the previous year. On the other hand, ICU-OD in 2020 was comparable to pre-pandemic levels and the reduction in 2021 was less pronounced than in 2022, indicating a maintained treatment capacity during the highest burden of the pandemic at the UH-NRW. ICU-OD started to decrease when the peak of COVID-19 patients admitted to the ICU was already over. Besides the strike in the second quarter of 2022, an increasing lack of adequate nursing staff is the most obvious explanation for this observation (17, 56).

There are probably many reasons for the increasing shortage of skilled workers in nursing professions. It could be shown, that the outbreak of the COVID-19 pandemic exerted significant mental burden on ICU healthcare staff as they experienced high levels of stress and burnout (57). Reduced ICU capacities in 2022 suggests that the pandemic continues to have an impact on the available staff operating ICU-beds in Germany, but presumably not mainly because hospitals still have to cope with COVID-19 patients or staff being absent due to (Covid-19) illness, but because of high rates of job dissatisfaction and burnout among healthcare workers. This might contribute significantly to the reduction of available ICU nurses in the post-COVID-19 period (58). The number of physicians working in hospitals in Germany increased by 1.2% in 2022 (59). However, as the area of activity of physicians in Germany is not recorded centrally, it cannot be concluded from this that the number of available ICU-physicians has also increased. Nevertheless, it can be assumed that the shortage of ICU-staff in 2022 was primarily due to a shortage of staff in the nursing professions (56).

### 4.4 Organ donation activities in Germany from 2019 to 2022

Comparing the numbers of DsBD, pDBD and uDBD from the OD-reports for the years 2019 to 2022 showed some remarkable results. In the first year of the pandemic, numbers in NRW, Germany and for the hospitals with different levels of care were comparably negatively affected. From 2021 on, however, the number of DsBD and pDBD increased in all level A hospitals together. In contrast, this increase was not detectable in the UH-NRW, although these are also level A hospitals. The nursing strike in 2022, which only affected university hospitals in NRW (55), could be an explanation for this finding. This indicates that the number of available nurses and consequently the number of operable ICU-beds was a crucial factor for the number of pDBD and consequently for the number of utilized donations in this cohort. There are seven level A hospitals in NRW in total (40). It can therefore be assumed that the numbers from the five level A hospitals in this study are representative for all level A hospitals in NRW.

But even a higher number of pDBD in all level A hospitals together in 2022 did not result in more utilized donations from these hospitals compared to the prepandemic period, indicating an increasing problem at the level of converting a pDBD into a uDBD. It can only be speculated that reduced ICU treatment capacity, resulting in a higher workload of ICU-staff had a negative impact on donor evaluation. At least, this was shown during the first wave of the pandemic (60).

Level C hospitals experienced a dramatic decline of DsBD, pDBD and uDBD from 2020 to 2022, probably because of an ongoing referrals of DsBD to hospitals with a higher level of care in times of reduced ICU capacities (39). Additionally, prepandemic studies could show, that level C hospitals had a significantly higher number of cases with an indicated but not performed diagnostic of braindeath in pDBD compared to university hospitals (33). The number of missed braindeath diagnoses, even though they were indicated, may have increased even more under the pressure of reduced intensive care capacities, although this assumption cannot be derived with certainty from the available data.

In total, there were 21.667 pDBD in Germany in 2022, compared to 23.948 in 2019, meaning that the pool of patients potentially eligible for a donation after braindeath has shown a notable decrease. By analogy with Tanner et al., who asked where the ST-segment-elevation myocardial infarctions (STEMI) have gone in the pandemic, given that their numbers fell by around 40% at the start of the pandemic (7), one could ask where all the potential donors have gone in the year 2022. The decline in STEMI could be explained by COVID-19 public health warnings, which may have inadvertently contributed to reduced contact of STEMI patients with hospitals or primary care physicians. Also, social distancing due to less contact with family members may have affected the ability to initiate hospitalization. Thus, changing patient behavior was a likely factor contributing to the decline in STEMI cases during the pandemic (7). However, it is unlikely that these explanations are applicable to pDBD in the year 2022. These patients are less likely to have avoided contact with a hospital of their own choice (21) and COVID-19 restrictions decreased in 2022. Although the number of deceased with a severe brain damage decreased, the overall mortality rate in Germany was higher in 2022 than in previous years (61). Consequently, more patients must have died for reasons other than a severe brain damage or more patients did not die in a hospital. However, due to the lack of centralized data collection and evaluation of services in the German health care system (5, 39) the question of why numbers of pDBD were lower in Germany in 2022 than in the prepandemic period cannot be answered with the available data.

### 4.5 Utilized donations during the first years of the COVID-19 pandemic in Germany

In the first years of the pandemic, donations in Germany remained stable in contrast to other countries. In fact, Germany had one of the best transplantation responses to the COVID-19 crisis in the early phases of the pandemic (14). Where transplant activity initially rapidly declined during the first wave of disease, it returned to baseline as the pandemic progressed, despite a higher burden of disease during the second wave (42). However, the total number of donors per million inhabitants and conversion rates in Germany are traditionally at a low level compared to other countries (40). In other words, it could be speculated that a poor performance was less likely to get worse. Additionally, Germany experienced fewer COVID-19 hospitalizations than other European countries during the first wave resulting in a smaller decline in transplant rates (42). Other countries also showed a larger decrease in DCD than DBD (9, 42). As DCD is not possible in Germany, this might explain—in addition to an overall higher ICU treatment capacity than other countries (52)—that there was no relevant reduction in utilized donations in Germany during the first years of the pandemic.

### 4.6 Limitations

This was a retrospective evaluation of potential donors at five university hospital in NRW, Germany and may thus not be transferable to the whole of Germany. Hospitals with a lower level of care may have suffered more from pandemic-related restrictions and this may have a significant influence on the number of potential and utilized donors. Additionally, university hospitals in NRW were affected by a strike of nursing staff in 2022, making it difficult, to compare numbers with other regions of Germany not affected by the strike.

The quality of the data from OD-reports may vary to an unknown extent. Despite legal regulations, the identification of a patient as a potential donor is still based on a partly subjective assumption by the treating physician. In addition, not every harvesting hospital provides sufficient data for the OD-reports, meaning that the response rate for the reports does not reach 100% for every year and region in Germany.

Relevant data was probably not collected in this retrospective analysis, such as the proportion of patients referred from other hospitals, the length of time from the onset of the disease to therapy, or the type of brain damage, all having a possible effect on the number of potential donors and the reasons against a donation.

The definition of a pDBD and the classification of the reason against a donation in pDBD are partly subjective and reasons against donation may include factors, that we were not able to access in this retrospective analysis. Whether a brain damage is severe enough to possibly lead to braindeath depends in part on the experience and assessment of the treating physician. The rate of refused consent may have been even higher, as consent to donation was probably not assessed at all in cases where preserved brainstem reflexes prevented a donation anyway.

## 5 Conclusion

The number of DsBD and pDBD decreased significantly in 2022 at five university hospitals in NRW, Germany, which was likely due to significantly reduced ICU treatment capacities. This reduction cannot be fully explained by the COVID-19 pandemic, as it occurred after its peak. In combination with a lower conversion rate, this resulted in a remarkable drop of utilized donations in 2022. The numbers from the organ donation reports of all harvesting hospitals in Germany indicate that the reduction in DsBD and pDBD were even more pronounced in hospitals of lower care. Together, this resulted in an overall reduction of utilized donations in Germany in 2022.

The pandemic and the subsequent shortage of health care professionals put light on the limitations of the German organ procurement program. Despite a comparably high supply of ICU beds, Germany was not capable to maintain donation numbers in the face of reduced ICU capacities. As the shortage of qualified staff in Germany is likely to remain a problem even after the end of the pandemic, this should prompt review of organ procurement processes and supply of staff and ICU beds. Otherwise, the number of utilized donations could decrease further in the future.

The results of this study indicate that with the current practice of the German organ procurement program, the number of organ donations appears to be strongly dependent on ICU capacities. In order to increase the volume of donations, Germany must therefore either increase ICU capacities again or restructure its organ procurement processes.

## Data availability statement

Datasets are available on request from the corresponding author. Requests to access these datasets should be directed to jan.englbrecht@ukmuenster.de.

## Ethics statement

The studies involving humans were approved by the Ethics Committee of the University of Muenster on July 18, 2023 (File Number 2021-801-f-S). The studies were conducted in accordance with the local legislation and institutional requirements. Written informed consent to participate in this study was not required from the participants in accordance with the national legislation and the institutional requirements.

## Author contributions

JE: Conceptualization, Data curation, Formal analysis, Investigation, Methodology, Resources, Visualization, Writing—original draft. DS: Data curation, Investigation, Writing—review & editing. JA: Data curation, Investigation, Writing—review & editing. MSc: Data curation, Investigation, Writing—review & editing. MSo: Data curation, Formal analysis, Investigation, Writing—review & editing.
